# A Role for the Action Observation Network in Apraxia After Stroke

**DOI:** 10.3389/fnhum.2019.00422

**Published:** 2019-12-20

**Authors:** Gloria Pizzamiglio, Zuo Zhang, James Kolasinski, Jane M. Riddoch, Richard E. Passingham, Dante Mantini, Elisabeth Rounis

**Affiliations:** ^1^Nuffield Department of Clinical Neurosciences, University of Oxford, Oxford, United Kingdom; ^2^Wellcome Centre for Human Neuroimaging, Institute of Neurology, University College London, London, United Kingdom; ^3^Social, Genetic and Developmental Psychiatry Centre, Institute of Psychiatry, Psychology and Neuroscience, King’s College London, London, United Kingdom; ^4^Cardiff University Brain Research Imaging Centre, Cardiff University, Cardiff, United Kingdom; ^5^Department of Experimental Psychology, University of Oxford, Oxford, United Kingdom; ^6^Research Centre for Motor Control and Neuroplasticity, KU Leuven, Leuven, Belgium; ^7^Brain Imaging and Neural Dynamics Research Group, IRCCS San Camillo Hospital, Venice, Italy

**Keywords:** apraxia, voxel-based lesion-symptom mapping, gesture production, gesture recognition, meaningless gesture imitation, superior temporal sulcus, action observation

## Abstract

Limb apraxia is a syndrome often observed after stroke that affects the ability to perform skilled actions despite intact elementary motor and sensory systems. In a large cohort of unselected stroke patients with lesions to the left, right, and bilateral hemispheres, we used voxel-based lesion-symptom mapping (VLSM) on clinical CT head images to identify the neuroanatomical correlates of the impairment of performance in three tasks investigating praxis skills in patient populations. These included a meaningless gesture imitation task, a gesture production task involving pantomiming transitive and intransitive gestures, and a gesture recognition task involving recognition of these same categories of gestures. Neocortical lesions associated with poor performance in these tasks were all in the left hemisphere. They involved the pre-striate and medial temporal cortices, the superior temporal sulcus, inferior parietal area PGi, the superior longitudinal fasciculus underlying the primary motor cortex, and the uncinate fasciculus, subserving connections between temporal and frontal regions. No significant lesions were identified when language deficits, as indicated via a picture naming task, were controlled for. The implication of the superior temporal sulcus and the anatomically connected prestriate and inferior parietal regions challenges traditional models of the disorder. The network identified has been implicated in studies of action observation, which might share cognitive functions sub-serving praxis and language skills.

## Introduction

Limb apraxia refers to a range of deficits in skilled action that are not consequences of motor weakness, sensory impairment, or lack of comprehension or coordination (Heilman and Rothi, 2003). Patients with the disorder have difficulties performing skilled actions, such as shaving or making a cup of tea. In stroke patients, limb apraxia can be demonstrated by impairments both when they use the affected and the unaffected hand. The syndrome is increasingly recognized as a predictor of poor functional recovery after a stroke that affects patients’ activities of daily living, with greater rates of patients with this disorder being dependent or ending up in nursing homes (Donkervoort et al., 2006; Bickerton et al., 2012). In addition to the motor impairments caused by this disorder, apraxia may worsen other cognitive impairments, such as aphasia, by compromising patients’ ability to communicate through gestures.

Traditional theories of the disorder have categorized praxis deficits according to errors made by patients in tasks involving (1) Imitation of both meaningless and meaningful gestures (e.g., asking a patient to copy meaningless hand or finger gestures or else to copy a familiar gesture, such as saluting), (2) Pantomiming of meaningful gestures or tool use (either intransitive, e.g., “show me how you stop traffic” or transitive gestures, e.g., “show me how you would brush your teeth, using a toothbrush in your hand”), and (3) Actual tool use (e.g., asking the patient to demonstrate the use of a torch) or in the performance of complex sequences of actions (e.g., asking the patient to make tea) (Leiguarda and Marsden, 2000; Donkervoort et al., 2006; Dovern et al., 2011). Whereas pantomime and object-use tasks pertain to deficits implicating conceptual (semantic) planning for meaningful gestures, imitation of meaningless gestures tests the implementation or production systems (Cubelli et al., 2000; Leiguarda and Marsden, 2000; Heilman and Rothi, 2003).

Most screening batteries for apraxia involve the use of pantomiming and imitation of meaningless hand gestures, because these tasks are particularly sensitive for detecting praxis deficits (Niessen et al., 2014; Buchmann and Randerath, 2017). This has formed the basis for their inclusion for testing praxis in the Birmingham Cognitive Screen (BCoS) (Bickerton et al., 2012; Humphreys et al., 2012).

Lesion-mapping studies investigating limb apraxia agree that left hemisphere damage plays a role in this disorder, implicating the fronto-temporo-parietal network (Mengotti et al., 2013; Buxbaum et al., 2014; Hoeren et al., 2014; Goldenberg and Randerath, 2015). They report a significant role for the inferior parietal lobe in tool-use pantomime and in imitation of meaningless gestures (Buxbaum et al., 2014; Hoeren et al., 2014; Dressing et al., 2018). However, there is no clear dichotomy between the two, as the neural correlates of pantomime are widespread (Daprati and Sirigu, 2006; Goldenberg et al., 2007; Price et al., 2010; Manuel et al., 2013; Goldenberg and Randerath, 2015).

Several factors could account for these findings. Lesion-mapping studies of apraxia have been limited by methodological issues, notably in the analysis methods used, and variability in the tasks used to study the disorder. There have been inconsistencies in the screening tools used to assess various subtypes of the disorder (Goldenberg, 2017). The lesion-symptom mapping methods employed have included the use of manual delineation of abnormal brain tissue, which can produce inconsistencies across operators (Gillebert et al., 2014). The use of dichotomized data, categorizing apraxia as being present or absent instead of including continuous scores, had meant that initial studies incorporated small numbers of patients.

The use of voxel-based lesion-symptom mapping (VLSM) has enabled the inclusion of much larger and unselected cohorts of patients in more recent studies (such as in Manuel et al., 2013; Buxbaum et al., 2014; Hoeren et al., 2014). The use of continuous, rather than dichotomized, apraxia scores has also allowed for a more fine-grained description of the neural correlates of praxis deficits by improving power in these analyses (Cohen, 1983). The variability caused by the inclusion of patients at various stages of recovery after stroke – from early subacute to chronic stages – is being mitigated by studying more homogenous cohorts of patients (Hoeren et al., 2014; Weiss et al., 2016).

An important factor that has been overlooked in several lesion-mapping studies of the disorder in the past has been the relationship of apraxia with other cognitive disorders, in particular, aphasia (Goldenberg and Randerath, 2015). Several studies report the co-occurrence of the two disorders, with little evidence of the presence of apraxia with no aphasia in right-handed patients (Selnes et al., 1991; Papagno et al., 1993; Weiss et al., 2016). This has become increasingly relevant in light of recent studies that indicate that pantomime of tool use, which is widely used in diagnosing this disorder (Buchmann and Randerath, 2017), might have a communicative role (Dressing et al., 2018; Finkel et al., 2018). A lesion-mapping study investigating apraxia and aphasia in left-hemisphere stroke patients distinguished between a network involving frontal, insular, inferior parietal, and superior temporal areas supporting language functions and lesions involving the sensorimotor, premotor, and parietal cortices associated with praxis tasks, with the inferior premotor area (BA44) co-localizing for both (Weiss et al., 2016). Another lesion-mapping study by Finkel et al. (2018) identified two putative networks sub-serving communication and motor functions when stroke patients pantomimed tool-use actions.

In this study, we make use of a large database of stroke patients that included both neuropsychological measures of praxis and imaging data, available from the patients’ clinical CT scans on admission. Previous lesion-symptom mapping studies of the disorder have used MR imaging because of the wide availability of analytic methods for lesion delineation in this imaging modality (Seghier et al., 2008), which can then be used to identify correlations between lesion and behavioral deficits (VLSM – Sperber and Karnath, 2018). Most of these studies investigated the disorder at the chronic stage (Manuel et al., 2013; Buxbaum et al., 2014), and some in the earlier stages after stroke (Hoeren et al., 2014). The advantage of investigating patients in the acute and subacute stages is that lesions directly relating to the stroke can be identified before changes such as atrophy (caused by post-stroke degeneration) take place (Lindberg et al., 2007). This is important when using automated lesion delineation techniques, as atrophy may affect the delineation of lesions.

Our aim was to investigate the neural correlates of deficits in praxis in a large cohort of subacute stroke patients who took part in the BCoS (Humphreys et al., 2012). The validity of the screening tasks for apraxia administered in the BCoS has been confirmed previously (Bickerton et al., 2012).

We used an automated CT processing toolbox, developed in our laboratory (fully described in Gillebert et al., 2014), which enabled lesion delineation for voxel-based lesion-mapping analyses to be performed. We conducted large-scale retrospective VLSM analyses (Bates et al., 2003) on a group of subacute unselected brain-damaged patients using continuous rather than descriptive cognitive scores of praxis from the BCoS (Humphreys et al., 2012).

## Materials and Methods

### Patients

The patients were recruited into the Birmingham Cognitive Screen project (BCoS), a multi-center clinical study investigating cognitive impairments after subacute stroke (patients were recruited from several stroke units across the West Midlands area of the United Kingdom). This study was approved by the National Research Ethics Service (NRES): Essex 1 Research Ethics Committee (REC) and local NHS trusts. Patients were included in the study if: (1) they were within 3 months of a confirmed first stroke and medically stable; (2) they were judged by the clinical team to be able to concentrate for at least 30 min to enable cognitive testing; (3) they had sufficient command of English to follow instructions; and (4) they were able to provide written informed consent to participate in the study (Bickerton et al., 2012). Hence, all the patients in this study had provided informed consent for the use of their neuropsychological and imaging data in the research.

The BCoS comprises the assessments of apraxia detailed below. Additionally, we included assessments of other cognitive domains, namely: attention, memory, language, and number processing. These data were supplemented by a CT head scan and demographic information, which was obtained from the patients’ clinical files.

Patients were excluded if they had no lesion visible on CT scan or had scans that were not adequate for further analyses (e.g., those not fulfilling the imaging criteria set out below). They were also excluded if they had ventricular enlargement documented in the report.

From an initial cohort of 484 patients who had taken part in the BCoS screening and had imaging available, a final sample of 387 sub-acute stroke patients who had both adequate imaging and a full set of praxis testing was included in this study. Patients with a first stroke in either their left, right, or both hemispheres were included to form an unselected, unbiased group of patients at the acute and subacute stages after stroke. Table 1 provides complete demographic information on the patient cohort. The group included 353 right-handed patients and 34 left-handed patients. Of the patients who were left-handed, four patients had right-hemisphere lesions, two patients had bilateral lesions, and 28 patients had left-hemisphere lesions. A total of 349 patients from the cohort had had an ischemic stroke, and 38 patients had had a hemorrhagic stroke.

**TABLE 1:** Patient demographics and imaging details (SD = standard deviation).

N Patients included	387		
Mean age	72.39 (ranging from 27–94; SD = 12.80)		
Gender	Females 200	Males 187	
Lesioned hemisphere	Left 202	Right 176	Bilateral 9
Mean time of assessment (days after stroke)	24.3 days (SD 17.1 days)	1 day	93 days
Mean years in education	11.4 years (SD 2.8 years)	5 years	24 years
Mean lesion size (mm^3^)	1.22 × 10^5^ (SD 1.4 × 10^5^)		

### Neuropsychological Assessments – Praxis Tasks

The cognitive assessment of the patients took place in hospital settings in the acute and sub-acute stage (≤3 months) post-stroke. The average time between stroke onset and test administration was 24.3 days (minimum = 1 day, maximum = 93 days), with 264 patients tested within 1 month after stroke. Neuropsychological testing was conducted using the BCoS (Humphreys et al., 2012).

The praxis tasks in the BCoS are aimed at assessing the cognitive processes subserving praxis, namely: (1) the input of visually conveyed gestures; (2) the coding of body part and position; (3) access to stored knowledge about the meaning of gestures; and (4) access to motor output transforming spatiotemporal concepts of gestures into motor commands (see Figure 1 in Bickerton et al., 2012; Humphreys et al., 2012).

**FIGURE 1:**
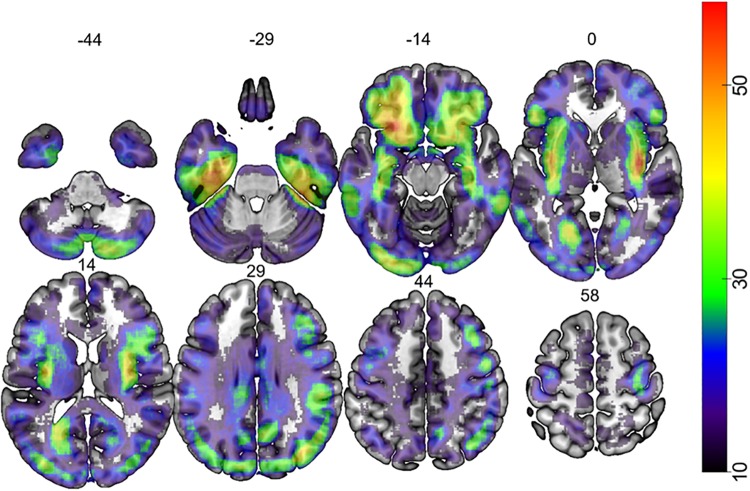
Map depicting the lesion overlap of 387 participants. The color bar indicates the number of patients that had lesion at each voxel. The number over each brain slice indicates the Z coordinate in MNI space.

In the current study, we used three of the BCoS praxis tests to assess the presence of apraxia: Gesture Production, Gesture Recognition, and Meaningless Gesture Imitation. The BCoS also includes an assessment of orientation in time and space, providing a brief measure of orientation in time, person, and place and of overall comprehension, which was used in our imaging analyses as a covariate of no interest to remove deficits in basic cognitive ability (which could be caused by other clinical conditions at early stages after stroke, such as delirium) as potential confounds.

A previous study examined the validity and reliability of the praxis tasks in the BCoS against existing screens and included the patient cohort reported here (Bickerton et al., 2012). The inter-rater reliability for praxis in this particular cohort of patients has been reported and published before, in Chapters 6 and 7 of the BCoS manual (Zwinkels et al., 2004; Humphreys et al., 2012).

According to criteria published previously (Humphreys et al., 2012), patients were considered apraxic if they scored below the previously published set cut-off score in at least one of these three praxis tasks. Table 2 lists the cut-off scores. However, for the purposes of the current study, the patients’ praxis scores were entered as a continuous variable for the imaging analyses. Each of the three praxis tasks is detailed below. Two of the tasks (Gesture Production and Gesture Imitation) required empty-handed execution of gestures to test conceptual and production deficits, respectively, according to traditional models of the disorder, without the confound of having the object-at-hand (Goldenberg, 2013a). Patients used their dominant hand or, if they had hemiparesis, their unaffected hand. A total of 266 patients used their left hand, and 121 patients used their right hand for the performance of all praxis tasks reported in this study.

**TABLE 2:** Age adjusted cut-off scores for praxis tasks used in this study.

	**Age range (Number of controls tested)**		
	**≤64**	**65–74**	**≥75**
	**(*N* = 34)**	**(*N* = 33)**	**(*N* = 33)**
Gesture Production	10	9	9
Gesture Recognition	5	5	4
Gesture Imitation	9	9	9

### Gesture Production

The Gesture Production task involved pantomiming a total of six gestures (three transitive, three intransitive) upon verbal command. The test included body-centered (salute, using a glass), non-body-centered (stop, using a salt cellar), repetitive (hitch-hiking, using a hammer), and non-repetitive (stop, using a glass) actions. All actions can be carried out as a single-step sequence. Patients were allowed a maximum of 15 s per item to respond and were asked to execute the action once. Two points were given for a correct and accurate gesture; 1 point for a recognizable but inaccurate gesture (e.g., including spatial and/or movement errors); 0 points were given for either no response after 15 s, an unrecognizable response or perseveration from previous gestures. The final sum score (maximum = 12) was used in the analyses.

### Gesture Recognition

In the Gesture Recognition Task, the examiner produced six actions, which patients had to recognize: three transitive (using a cup, using a key, using a lighter) and three intransitive (come over, good, goodbye) actions. As the examiner showed each gesture, the patients had to select the action being performed from a multiple-choice list, which included four alternative responses for each action, in writing. The four alternatives for each action corresponded to: (1) the correct action (e.g., using a lighter); (2) a semantically related action (using a match); (3) a visually related action (using a gun); and (4) an unrelated action (using a torch). The patients were allowed a maximum of 15 s per item to respond by pointing to their chosen statement, and they were given one point for each correct response. The final sum score (maximum = 6) was used in the analyses.

The data from both transitive and intransitive gestures in these tasks were entered together as a composite measure. Hence this study does not report differences between the two.

### Meaningless Gesture Imitation

The patients were asked to copy four meaningless gestures presented by the examiner. Two of these gestures involved a sequence of two hand positions in relation to the head, and the other two involved a single finger position. This task contrasted the indirect route to action production (i.e., imitating meaningless gestures) with “lexical” action recognition and production to name (see Bickerton et al., 2012). Three points were given for a gesture that was correctly and precisely imitated after the 1st presentation; two points if the gesture was correct and precise after the 2nd presentation; 1 point if patients made only one error after the 2nd presentation (e.g., incomplete movement sequence, incorrect spatial relationship between hand and head, or incorrect finger/hand position); 0 points if patients made more than one error, gave no response or showed perseveration from previous item(s) after the 2nd presentation. The final sum score (maximum = 12) was used in the analyses.

Table 2 gives the praxis tasks cut-off scores based on the 5th percentile across age groups (from Humphreys et al., 2012). We report the rates of praxis deficits according to these cut-off scores in the section “Results.”

### Picture Naming

The Picture-Naming task was used to control for language deficits in our study. The task involves asking patients to name objects that the examiner shows them a picture of. There were 14 objects that patients had to recognize and name. These were: bell, peas, grape, umbrella, raspberry, colander, leak, stopwatch, bat, pineapple, chisel, tiger, hook, and spanner. Patients scored one point for each correct naming, with a potential total score of 14.

### Imaging and Lesion Analysis

#### CT Data Acquisition

CT scans were acquired as part of the patients’ clinical assessment during their hospital admission. For the 387 patients included in this study, the average time between the stroke and CT scan acquisition was 4.4 days (Minimum = 0 days, Maximum = 64 days; Standard Deviation of 11 days, with more than 80% of cases scanned within 1 week).

The study used standardized CT imaging protocols, as follows. The scanners used were a Siemens Sensation 16 and a GE Medical System LightSpeed 16 and LightSpeed Plus. The images covered the whole brain, with slices aligned along the AC-PC plane and an in-plane resolution of 0.5 × 0.5 mm^1^ and a slice thickness varying between 4 and 5 mm. A CT database of more than 500 patients with acute/subacute stroke was available, together with their clinical and demographic data, as well as a completed battery of neuropsychological tests from the BCoS (Humphreys et al., 2012). Patients with inappropriate CT scans were excluded from the study: these were patients with a CT scan in which a shunt was visible or patients in whom the field of view did not encompass the head (*n* = 127) (Gillebert et al., 2014).

#### Automated Lesion Delineation Method

We implemented an automated toolbox for pre-processing and lesion mapping of CT brain scans (Gillebert et al., 2014). This procedure, fully described in Gillebert et al. (2014), involved the normalization of CT images from stroke patients to template space (Rorden et al., 2012a). Areas of hypo- or hyper-intensity, corresponding to ischemic or hemorrhagic stroke, respectively, were defined by voxel-wise comparisons with a group of control CT images. The validation and effectiveness of this approach were demonstrated both by visual inspection using CT images in sub-samples of stroke patients from the same dataset as in this study (CT image database collected for the Birmingham University Cognitive Screen, see text footnote 1) and by using simulated lesions. Both checks are reported in a previous study (Gillebert et al., 2014).

According to this method, CT scans were pre-processed using SPM8 (The Wellcome Trust Centre for Neuroimaging, London, United Kingdom), and lesion delineation was performed using in-house software written in Matlab (The MathWorks, Natick, MA, United States). Firstly, threshold-based clustering at 0.1% maximum intensity was implemented to remove irrelevant signals (Batenburg and Sijbers, 2009). The resulting CT images were spatially aligned to a template using the co-registration tool in SPM8. CT image intensity was then transformed using an invertible formula to emphasize the contrast between cerebrospinal fluid and parenchyma (Rorden et al., 2012a).

The converted CT images were then warped to MNI space using a CT template (Rorden et al., 2012b). Firstly, a normalization function was used to calculate and apply a 12-parameter affine transformation that maximized the alignment to the template. The distribution of all image intensities was then calculated to create masks of the brain and the ventricles that were applied to generate skull-stripped images. These were then normalized and resliced at a 1-mm isotropic resolution using a large bounding box that included both the cortex and the cerebellum. The normalized CT images were smoothed with a 4-mm FWHM Gaussian filter (Salmond et al., 2002; Stamatakis and Tyler, 2005) according to the assumption of random field theory used in the statistical analysis (Worsley, 2003).

The lesion of each stroke patient was automatically identified using a voxel-based outlier detection procedure based on the Crawford-Howell parametric *t*-test for case-control comparisons (Crawford and Howell, 1998; Crawford et al., 2009). An outlier t-score map was generated using this test that coded the degree of abnormality of each voxel intensity based on a comparison to the normal range from control CT scans. These t-score maps were thresholded to generate binary lesion maps in MNI space (Gillebert et al., 2014) that were used to perform VLSM analyses.

#### Voxel-Based Lesion-Symptom Mapping

The lesion maps obtained from the aforementioned procedure underwent VLSM analyses to identify the neural underpinnings of praxis deficits after stroke, based on the analysis toolbox provided by Bates et al. (2003)^4^. The behavioral results for each of the praxis tasks available from 387 patients were entered into separate VLSM analyses as the variable of interest, with additional covariates of age, handedness, total lesion volume, and assessment of orientation in time and space to control for these confounding factors (Gillebert et al., 2014; Chechlacz et al., 2015). We added an assessment of orientation in time and space based on correlations of deficits in this generic cognitive domain with praxis.

A linear model was fitted at each voxel, relating the unique score for each praxis task to lesion intensity (0 for no lesion; 1 for lesion). Tests were confined to those voxels in which at least 10 patients had a lesion. Only voxels that reached the false discovery rate (FDR) threshold of *p* < 0.05 were considered significant.

The use of CT imaging did not allow a clear segmentation of gray and white matter as is usually performed in VLSM analyses of MRI data. However, this has been used in CT in previous publications on neglect and attention (Gillebert et al., 2014; Chechlacz et al., 2015).

**FIGURE 2:**
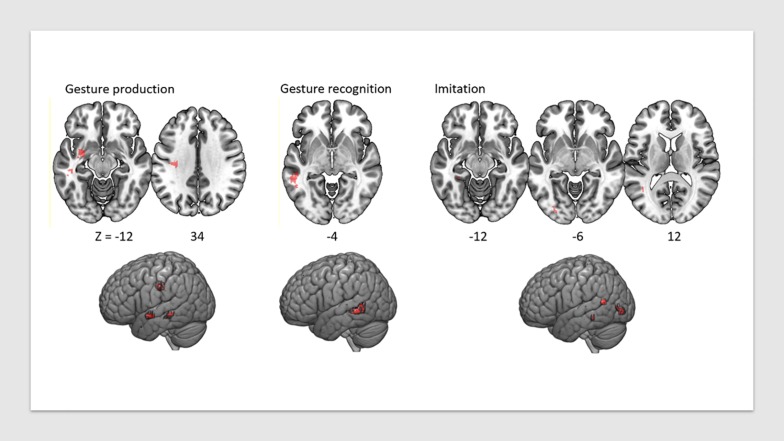
VLSM map of lesions associated with praxis deficits in each of the three tasks, FDR-corrected at *p* < 0.05, displayed on a T1 anatomical template in MNI space.

The anatomical localization for significant regions (FDR-corrected at *p* < 0.05) was identified based on the multi-modal parcellation of human cerebral cortex provided by the Human Connectome Project (HCP) (Andreas, 2016; Glasser et al., 2016). The anatomical localization of regions located within white matter tracts was based on the Catani Atlas of Human Brain Connections (Thiebaut de Schotten et al., 2011). The interpretation of our results was supported by the expertise of an anatomist (Prof. R. E. Passingham). Figures 1, 2 were created using the template at MRICroGL^2^.

## Results

### Behavioral Results

The behavioral results from individual praxis tasks and comparisons with other cognitive functions were used to identify the prevalence of deficits in each subtask in this cohort of patients. *Note that this analysis was not used to inform the lesion-mapping analyses reported below.* Instead, the behavioral data for each task were entered as a continuous variable. The reason for reporting the behavioral results below was to provide an indication of the number of patients who were deemed to be performing below the cut-off for praxis on these screening tasks. This was not used to inform our imaging analyses.

In the behavioral analyses, cut-off scores for normal performance (two standard deviations below the mean of age-matched healthy controls) were 11.5 on Gesture Production, 5.8 Gesture Recognition, and 11.5 in Imitation, based on normative data published previously (see Table 2 and Chapter 6 and 7 of Humphreys et al., 2012). Based on these criteria, 204 out of 387 patients performed abnormally on Gesture Production, 248 out of 387 on Gesture Recognition, and 252 out of 387 Imitation (on average, 235 patients out of 387 scored below range for apraxia). Of the left-handed patients, 12 out of 28 patients with left-hemisphere damage had no praxis deficits, whereas 16 patients with left-hemisphere damage scored below the cut-off in at least two of the praxis tasks, indicating they were most likely left-hemisphere dominant (Goldenberg, 2013b).

The average patient results on the three praxis tasks are outlined in Table 3.

**TABLE 3:** Patients’ average results in the three praxis tasks.

**Task**	**Mean**	**SD**	**MIN**	**MAX**
Gesture production	10.21	2.60	0	12
Gesture recognition	4.88	1.11	0	6
Gesture imitation	9.03	2.74	0	12

In addition to praxis scores, we computed patients’ general orientation in time and space and aphasia (using a picture-naming task from the BCoS). A total of 63 out of 387 (16%) of patients performed below the cut-off score for the orientation task, and 212 patients out of 387 (55%) performed below the cut-off score for picture naming, indicating language deficits adjusted for age.

We ran correlation analyses to identify whether our covariates of no interest were significantly correlated with a composite measure of apraxia, incorporating the scores of each of the three praxis tasks. Orientation in time and space correlated significantly with the composite Apraxia score (*r*_387_ = 0.396, *p* < 0.0001), as did lesion size (*r*_387_ = −0.120, *p* = 0.018) and age (*r*_387_ = −0.200, *p* < 0.0001).

### Imaging Results

Lesion overlap is shown in Figure 1. Figure 2 shows the lesion-symptom maps for each of the three tasks, FDR-corrected at *p* < 0.05, in axial and rendered views; Table 4 provides the coordinates for each area and each task.

**TABLE 4:** Coordinates of lesion-symptom mapping results, FDR-corrected at *p* < 0.05, based on HCP (Andreas, 2016; Glasser et al., 2016) and Catani white matter (Thiebaut de Schotten et al., 2011) atlases.

				**MNI coordinates**		
**Praxis**		**Volume**				
**tasks**	**Areas**	**(mm^3^)**	***t-*value**	**X**	**Y**	**Z**
Gesture Production	L Superior temporal Sulcus (STSv posterior)	224	3.992	−50	–36	−12
	L Uncinate Fasciculus L Superior Longitudinal Fasciculus	463	4.413	−28	–4	−16
		558	4.051	−34	–25	31
Gesture Recognition	L Superior temporal Sulcus (STSv posterior)	508	4.240	−54	–44	−6
Gesture Imitation	L Prestriate (V4)	272	5.739	−29	–88	−8
	L Superior Temporal Sulcus (PGi)	54	4.966	−42	–56	13
	L Inferotemporal Cortex (ParaHippocampal Area 2)	48	5.129	−32	–39	−15

Deficits in the Gesture Production task were associated with lesions in a network of areas involving the left superior temporal sulcus (*x* = −50, *y* = −36, *z* = −12; *t* = 3.99), the left uncinate fasciculus (*x* = −28, *y* = −4, *z* = −16; *t* = 4.41) (which connects the temporal lobe with the inferior frontal cortex including Broca’s area), and the white matter beneath the left primary motor cortex, within the superior longitudinal fasciculus (*x* = −34, *y* = −25, *z* = 31; *t* = 4.05). The lesions identified disconnections between the temporal and parietal lobes with the frontal lobe, leading to impairment in converting gestures into motor commands.

Deficits in the Gesture Recognition task revealed significant associations with lesions in the left superior temporal sulcus (*x* = −54, *y* = −44, *z* = −6; *t* = 4.24).

Finally, regions significantly associated with the meaningless gesture imitation task comprised the left visual striate and pre-striate cortices (*x* = −29, *y* = −88, *z* = −8; *t* = 5.74), PGi parietal area (*x* = −42, *y* = −56, *z* = 13; *t* = 4.97), and parahippocampal area (*x* = −32, *y* = −39, *z* = −15; *t* = 5.13). We report the results for all patients combined in Table 4.

Subgroup analyses were performed to identify lesions pertaining to right- versus left-handed patients with right- versus left-hemisphere lesions, separately. Only the analyses pertaining to right-handed patients with both left- and right-hemisphere lesions combined revealed significant results (FDR-corrected at *p* < 0.05). No significant results were identified in the other subgroups. Nevertheless, we identified the lesion locations at *p* < 0.005 uncorrected for left-, followed by right-hemisphere lesions in right-handed patients, which are reported in section “Subgroup VLSM Analyses” of the Supplementary Material. Of note, unlike other reports (Goldenberg, 2013b), in our data, there were no significant differences in performance of the BCOS praxis tasks between subgroups of patients, as reported in this dataset previously (Bickerton et al., 2012; Humphreys et al., 2012).

A follow-up analysis was performed to identify lesion-symptom mapping that isolated praxis deficits from screened in the BCoS from language (picture naming). This was done by re-running the VLSM analyses outlined above with scores from the Picture Naming task in the BCOS (Humphreys et al., 2012) as an additional covariate. No significant results were found in this analysis.

We explored this result further by correlating the separate praxis with the picture-naming task. Picture Naming significantly correlated with Gesture Production (*r*_387_ = 0.501, *p* < 0.0001), Gesture Recognition (*r*_387_ = 0.368, *p* < 0.0001), and Meaningless Gesture Imitation (*r*_387_ = 0.407, *p* < 0.0001). Moreover, we implemented VLSM analyses for Picture Naming. The results identified the superior temporal gyrus and are provided in the Supplementary Material (section “VLSM Results of Picture Naming Task”).

## Discussion

We conducted VLSM analyses for apraxia based on a large cohort of acute and subacute stroke patients. A validated battery of cognitive tasks for praxis (BCoS) was used (Bickerton et al., 2012) and analyzed alongside clinical CT images in which stroke lesions were automatically delineated. Our findings relate specifically to the early stages after stroke. Left, right, and bilateral hemisphere lesions were included in a VLSM analysis, in which the patients’ scores in three praxis tasks from the BCoS were entered as continuous variables, creating an unbiased data sample.

Our results confirmed that deficits leading to apraxia result from left-hemisphere lesions (Goldenberg, 2013a). The lesion locations identified involved a network of areas comprising extrastriate visual areas, superior and medial temporal gyri, inferior parietal and inferior frontal areas, and white matter connections between the latter. As in recent VLSM studies of apraxia, our findings challenge traditional theories, which describe a prominent role of the parietal lobe in the disorder (Goldenberg, 2014). We identified instead ventral stream regions that pertain to the action-observation network. We discuss our results in relation to previous studies of apraxia, drawing parallels with the literature on language disorders after stroke. The last section highlights the implications of using clinical CT imaging in lesion-symptom mapping of apraxia.

### Neural Correlates of Apraxia Identified in Our Study

Our results identified an association of left-hemisphere lesions affecting the superior and medial temporal areas with all praxis tasks, namely gesture production, recognition, and meaningless gesture imitation. In addition, damage to the underlying white matter connections between the temporal cortex and the inferior frontal gyrus (the uncinate fascicle), as well as the superior longitudinal fasciculus underlying the primary motor cortex (Pandya et al., 2015), were associated with deficits in gesture production. Damage in the inferior parietal region PGi (Andreas, 2016; Glasser et al., 2016), prestriate, and parahippocampal area 2 (as identified in the HCP atlas; fusiform area, in other atlases) were associated with deficits in meaningless gesture imitation.

Voxel-based lesion-symptom mapping studies of apraxia report wide networks of brain regions in the disorder, parallelling ours. These include inferior frontal (Pazzaglia et al., 2008), parietal, and temporal (Buxbaum et al., 2014; Hoeren et al., 2014) and also subcortical areas (Pramstaller and Marsden, 1996; Haaland et al., 2000; Leiguarda and Marsden, 2000). An important factor determining the outcome of patient studies relates to the tasks used to elicit conceptual and production deficits in apraxia, as well as the imaging modalities used to study these brain functions. We discuss the impact of these in the sections below.

### Traditional Brain Networks Identified in Apraxia and the Role of Tasks Used in Understanding the Neural Correlates of the Disorder

Lesions of the parietal lobe, particularly affecting the dominant hemisphere, have traditionally dominated neuropsychological models of apraxia (Liepmann, 1908, 1920). Much of our understanding of the role of parietal areas in action has come from anatomical and physiological studies of non-human primates. A dorsal visual stream has been subdivided into dorso-dorsal and ventro-dorsal streams, subserving motor representations allowing the implementation of reach and grasp actions, respectively (Rizzolatti and Matelli, 2003; Daprati and Sirigu, 2006). The ventral stream, which was originally proposed to mediate perceptual information (Goodale and Milner, 1992), has also been shown to play a role in the selection of actions (Milner and Goodale, 2008; Weiller et al., 2009; Rijntjes et al., 2012). Recent literature suggests there are connections between the two, supporting a role for ventral stream structures in both action observation and object use (Borra et al., 2010; Ramayya et al., 2010; Passingham et al., 2014; van Polanen and Davare, 2015).

#### Parietal Cortex Contribution to Apraxia

The role of the inferior parietal cortex in limb apraxia has been reported in studies that used both real object-use tasks (Goldenberg and Hagmann, 1998; Osiurak et al., 2008; Goldenberg and Spatt, 2009) and pantomime of object use (Buxbaum et al., 2014; Hoeren et al., 2014). Functional neuroimaging studies report a prominent role for the left inferior parietal cortex in the actual use of objects (Lewis, 2006; Osiurak and Badets, 2016; Reynaud et al., 2016). Our study did not involve the use of functional neuroimaging, and the tasks used for screening for apraxia involved pantomime of both transitive (with objects) and intransitive (with no objects) gestures. In particular, it did not include the use of real objects.

The lack of significant lesions in inferior parietal areas in our pantomime tasks could be due to task-related factors (reported below) and the imaging modality used (namely lesion-symptom mapping rather than fMRI, reported in greater detail in sections “Materials and Methods,” “Settings Used for Our Voxel Based Lesion Symptom Mapping” of the Supplementary  Material, and “Interpretation of Our Imaging Results Based on CT Imaging”).

In relation to the former, it is noteworthy that there are anatomical connections between the superior temporal areas identified in our study and the inferior parietal areas reported in non-human primates (Rozzi et al., 2006). One possibility is that an effect of a lesion in the superior temporal sulcus could be to disconnect flow of information relating to biological motion (see below) from the inferior parietal cortex. This could elicit behavioral deficits in tool use. Lesion-mapping studies are descriptive. Unlike functional neuroimaging studies, they do not give an appreciation of how lesions in one area might impact activation or function in another area connected to it.

#### Role of the Temporal Cortex in Apraxia

There is an increasing amount of evidence for a communicative component to pantomiming gestures, even those that pertain to object-use. A lesion-symptom mapping study involving pantomiming of object-use identified two networks implicated in the task: a “posterior” network of brain regions, comprising inferior parietal and dorsal stream areas, representing the motor aspects of object use and an “anterior” network of brain regions, comprising inferior frontal and temporal areas, relating to the communicative components of the task (Finkel et al., 2018). The Gesture Recognition task in the BCOS requires comprehension of gestures and what they represent when choosing among a multiple-choice set of options in writing. What is more, the scores we obtained from Gesture Recognition and Gesture Production tasks combined both transitive and intransitive gestures, possibly emphasizing a role for communication as in Finkel et al.’s (2018) study. The lesions identified in our tasks were located predominantly in superior temporal rather than parietal areas, corresponding to the “anterior” network, which was attributed to communication in Finkel et al’s., 2018 study.

Nevertheless, our results on the Meaningless Gesture Imitation task, which did not require any verbal comprehension, also implicated both the superior and infero-temporal cortex, as well as inferior parietal area PGi. The study by Buxbaum et al. (2014) also identified lesions in the posterior temporal lobe and temporo-occipital areas as significant both in gesture representations of tools and in abstract movement representations when tested with meaningless gesture imitation. Both our results and theirs challenge the traditional model of apraxia in which the parietal lobe plays a central role, revealing the involvement of a wider network that comprises the left temporal lobe in the disorder (Goldenberg, 2009).

In the sub-sections below, we argue for a possible role of the temporal cortex in understanding action intentions, either through comprehension or through action observation.

##### A role for the temporal cortex in praxis and comprehension

In our study, we found no voxels pertaining to apraxia alone when covarying for language deficits measured using a Picture Naming task. Moreover, the two deficits co-existed in approximately 50% of our patient cohort. Lesions involving superior temporal areas, identified in Gesture Production, were also present in Picture Naming. Taken together our findings suggest that the two deficits might overlap (Goldenberg and Randerath, 2015; Finkel et al., 2018). In another study by Weiss et al. (2016), praxis and language were differentiable.

One reason for the discrepancy between our and Weiss et al.’s (2016) results could relate to the behavioral tasks used in this study. The tasks used in our study were part of a cognitive screening program developed to test stroke patients (BCOS, Humphreys et al., 2012) in which language is tested using Picture Naming. This task involves the naming of a large number of graspable objects (Bub et al., 2018). Previous studies using fMRI have identified a role for dorsal stream structures in identifying manipulable objects (Chao and Martin, 2000; Creem and Proffitt, 2001). There is evidence that naming manipulable objects influences actions (Bub et al., 2018; Masson, 2018). One possible explanation of our inability to differentiate between these two disorders in our data might relate to the fact that ventral stream networks to “name” and “use” objects may overlap (Mahon et al., 2007). Another possibility relates to the fact that both Gesture Recognition and Production tasks in the BCOS involve comprehension and that this may overlap with language functions (Goldenberg and Randerath, 2015). Our measure of these praxis tasks combined transitive and intransitive gestures, which have been shown to test for communication (Johnen et al., 2016; Dressing et al., 2018; Finkel et al., 2018).

Nevertheless, these factors still fail to explain the fact we identified the superior temporal gyrus in a meaningless gesture imitation task that involved no communication. We outline below a possible explanation for this latter result.

##### Regions identified in our task that form part of the action observation network

The involvement of the superior temporal area in the gesture imitation task in our study, which did not involve any verbal or semantic interpretations, parallels the roles described for these areas in action observation, which have been identified in non-human primates.

Studies have demonstrated the presence of cells in the superior temporal sulcus that code for action observation and are sensitive to biological motion stimuli (Jellema and Perrett, 2003; Barraclough et al., 2009). This region is anatomically connected to inferior parietal regions, which in turn connect to central premotor areas (Rozzi et al., 2006; Borra et al., 2008). The latter network of areas has been described as the “mirror neuron” network (Bonini et al., 2011), which is involved in understanding actions. Similar areas have been described in human fMRI studies, with evidence that the inferior parietal region is activated when healthy subjects are required to understand the meaning of gestures (Passingham et al., 2014) or when experts are asked to observe skillful actions that are familiar (Calvo-Merino et al., 2005). In our study, both the prestriate and inferior temporal cortices were involved in the imitation of meaningless gestures. This could relate to the role of the inferior temporal cortex in the discrimination between shapes (Huxlin et al., 2000). It may be that patients have to understand the shape of the hand that has to be copied.

A patient study by Achilles et al. (2016) provides some support for the above. Left-hemisphere stroke patients with and without apraxia were asked to rate the familiarity of meaningless gestures, which they imitated. Patients with apraxia were found to have better performance when copying meaningless gestures that were judged as being familiar by the whole patient cohort, suggesting that they were able to recognize familiarity in meaningless gestures.

Our results support a role for temporal lobe and prestriate areas in understanding the meaning of actions in meaningless gesture imitation tasks, even when language functions are not implicated (Buxbaum et al., 2014; Passingham et al., 2014). This might provide a non-verbal network sub-serving both the understanding of action intentions and communication.

##### “Domain-general” and “domain-specific” deficits after stroke and interpretation of our lesion-mapping results

The presence of similar areas sub-serving functions such as praxis and language skills might indicate that their involvement in these could be generic to both tasks (Geranmayeh et al., 2014). This has been demonstrated in the case of parietal lobe involvement, which is implicated in a large range of cognitive functions (Humphreys and Lambon Ralph, 2015). In the language literature, the parietal cortex has been shown to influence both “domain-general” and “domain-specific” deficits. An example of the former is the “Multiple Demand” system, which exerts top-down control on a wide range of tasks and involves processes such as cognitive flexibility, behavioral inhibition, and attentional control (Duncan, 2010; Hampshire et al., 2012).

The same is likely to be true for the role of the temporal lobe in praxis. Based on the literature, the role of the temporal cortex in praxis may be “domain specific”, in providing knowledge of tool function (Campanella et al., 2010; Buxbaum et al., 2014; Hoeren et al., 2014), or “domain general”, in understanding action meaning and “theory of mind” (Allison et al., 2000; Saygin, 2007). The former system may be used for naming and using tools (Mahon et al., 2007), whilst the latter system would be used for understanding others’ intentions through actions and non-verbal communication cues (Allison et al., 2000; Finkel et al., 2018).

Our study, like others, highlights a relationship between language and apraxia (Goldenberg and Randerath, 2015). However, we cannot draw conclusive evidence of the influence of one on the other. Some authors have tried to achieve such a differentiation with novel imaging analyses in lesion-symptom mapping, allowing the subtraction of one effect from the other (Dressing et al., 2018). However, to formally differentiate the relative contribution of the temporal lobe between the two cognitive domains, a systematic comparison between language and praxis skills would require more dedicated tasks, which would include tasks for biological motion targeted at differentiating between speech and hand gestures. This would need to be supplemented with converging evidence from fMRI and lesion-mapping techniques (Mahon et al., 2007).

#### Interpretation of Our Imaging Results Based on Clinical CT Imaging

This study is one of a few to have implemented lesion-symptom mapping techniques on the clinical CT scans of a retrospective cohort of stroke patients (Rorden et al., 2012b; Gillebert et al., 2014; de Haan and Karnath, 2018). Clinical CT is the imaging method of choice in patients admitted to hyperacute stroke units in the United Kingdom.

Recent advances (Ripolles et al., 2012; Rorden et al., 2012b) have made the identification of both ischemic and hemorrhagic lesions possible on the same CT scan (Chawla et al., 2009; Gillebert et al., 2014). The lesion delineation technique we used compares CT image intensity from a single patient with a group of images from control participants to identify outlier voxels (Crawford et al., 2009; Gillebert et al., 2014). In effect, this approach resembles the analysis of MR images (Stamatakis and Tyler, 2005). The use of standardized preprocessing techniques for CT (Rorden et al., 2012a) allowed us to obtain comparable results, in terms of lesion localization, to those reported in MRI studies (Buxbaum et al., 2014; Hoeren et al., 2014). Nevertheless, our lesion sizes and the number of patients required to obtain these results did differ significantly from lesion-mapping techniques that have used MRI (Manuel et al., 2013; Buxbaum et al., 2014; Weiss et al., 2016). This may have occurred due to the following methodological caveats. (1) The use of automated lesion delineation in our study may have underestimated lesion sizes, particularly for ischemic strokes, which are often difficult to detect on CT. The technique might benefit from more refined information that could be provided with complementary perfusion CT (Wing and Markus, 2019), which was not available at the time of data collection. (2) A study investigating the impact of sample size on the reproducibility of lesion-symptom mapping results (Lorca-Puls et al., 2018) reported striking differences in terms of either under- or over-estimated effect sizes. An additional shortcoming of lesion-symptom mapping techniques called “the partial injury problem” (Rorden et al., 2009) is that they may fail to consider the contribution of anatomically distributed areas in producing a behavioral deficit. This is because patients may present with different lesions in a distributed network, for which mass univariate analyses may miss the critical regions involved, due once again, to low statistical power (Herbet et al., 2015; Gajardo-Vidal et al., 2018). Some authors have proposed ways of mitigating the biological constraints of lesion distributions with the use of multivariate pattern analysis techniques (Smith et al., 2013; Mah et al., 2014). (3) Patient selection: although we tried to obtain an unbiased data-sample, the majority of our patients had strokes affecting the middle cerebral artery, with lesions located in the convexity of the hemisphere. This led to low numbers of patients with more superior lesions, probably reducing the statistical power to detect effects in these cortical regions (Kroliczak and Frey, 2009; Agnew et al., 2012; Buxbaum et al., 2014). (4) Lesion localization: the use of CT imaging had the caveat of requiring different anatomical atlases for gray and white matter localization. The review by de Haan and Karnath (2018) outlines significant differences in the interpretation of lesion mapping results based on which atlas is used for anatomical localization. Atlases such as the AAL (Tzourio-Mazoyer et al., 2002) and Harvard-Oxford atlases (Desikan et al., 2006), which are widely available in statistical analysis packages, under-represent the number of cortical areas (Van Essen et al., 2012). To avoid the mislabeling of areas (Passingham and Rowe, 2015), the anatomical localization of significant regions in this study were identified using separate atlases for white and gray matter regions (see section “Materials and Methods” and Table 4, above). For localization of gray matter areas, we selected to use a more detailed atlas, namely the HCP atlas (Andreas, 2016; Glasser et al., 2016).

## Conclusion

We have conducted a lesion mapping study on praxis deficits with the largest cohort studied to date. The patients were in the early stages after a stroke (Bernhardt et al., 2017). Our results suggest an important role for temporal lobe structures in the disorder. This area was not only implicated in the knowledge of tool functions when testing patients on pantomime tasks but was also present in the imitation of meaningless gestures. This finding concurs with other VLSM studies of the disorder in stroke (Buxbaum et al., 2014; Hoeren et al., 2014) as well as with previous literature involving praxis deficits in neurodegenerative disorders (Crutch et al., 2007; Johnen et al., 2016).

The implication of ventral stream areas in praxis, even when no object recognition is required, such as in the meaningless gesture imitation task, has been largely overlooked (Goldenberg, 2014). It is likely that the network implicated in apraxia evolved to sub-serve parallel functions for praxis and language in humans (Badets and Osiurak, 2017). New tasks are being developed that provide evidence that skillful tool use may support linguistic abilities (Brozzoli et al., 2019). Our results support recent studies designed to use action observation tasks for the rehabilitation of this devastating disorder (Pazzaglia and Galli, 2019). Further work is required to identify the granularity of the contributions of the temporal lobe and its connections in praxis and language deficits in patients with stroke and neurodegenerative conditions.

The adoption of analysis techniques borrowed from MRI (Seghier et al., 2008) that help the automated normalization into standard space and, therefore, inter-individual comparisons of CT images provides a window of opportunity for lesion-symptom mapping in larger patient cohorts (Gillebert et al., 2014). This will pave the way for a better understanding of cognitive deficits after stroke, such as apraxia.

## Data Availability Statement

The datasets generated for this study are available on request to the corresponding author and contingent on the approval of sharing this dataset by the BCOS team and local ethics committee.

## Ethics Statement

The studies involving human participants were reviewed and approved by the National Research Ethics Service (NRES): Essex 1 Research Ethics Committee (REC). The patients/participants provided their written informed consent to participate in this study.

## Author Contributions

ER conceptualized the study with DM. DM provided the data analysis techniques, which GP and ZZ implemented. JR was part of the original BCOS team who collected the data and together with the late Professor Humphreys provided access to it to complete this study. ZZ re-analyzed data with covariates of no interest (of aphasia and neglect) and created the Figure 1. JK and ZZ created the supplementary figures. JK and RP provided the anatomy and atlas support. ER and RP wrote up the manuscript. All authors reviewed and edited the manuscript.

## Conflict of Interest

The authors declare that the research was conducted in the absence of any commercial or financial relationships that could be construed as a potential conflict of interest.

## Footnotes

1 https://www.mccauslandcenter.sc.edu/mricrogl

2 http://aphasialab.org/vlsm
