# Supplementary material for: A Role for the Action Observation Network in Apraxia After Stroke
Source: Front Hum Neurosci. 2019 Dec 20;13:422. doi: 10.3389/fnhum.2019.00422 (PMC6933001; doi:10.3389/fnhum.2019.00422)
Supplement: Supplementary file 1 [file Data_Sheet_1.docx]

Supplementary Material:

1. *VLSM results of Picture Naming Task*


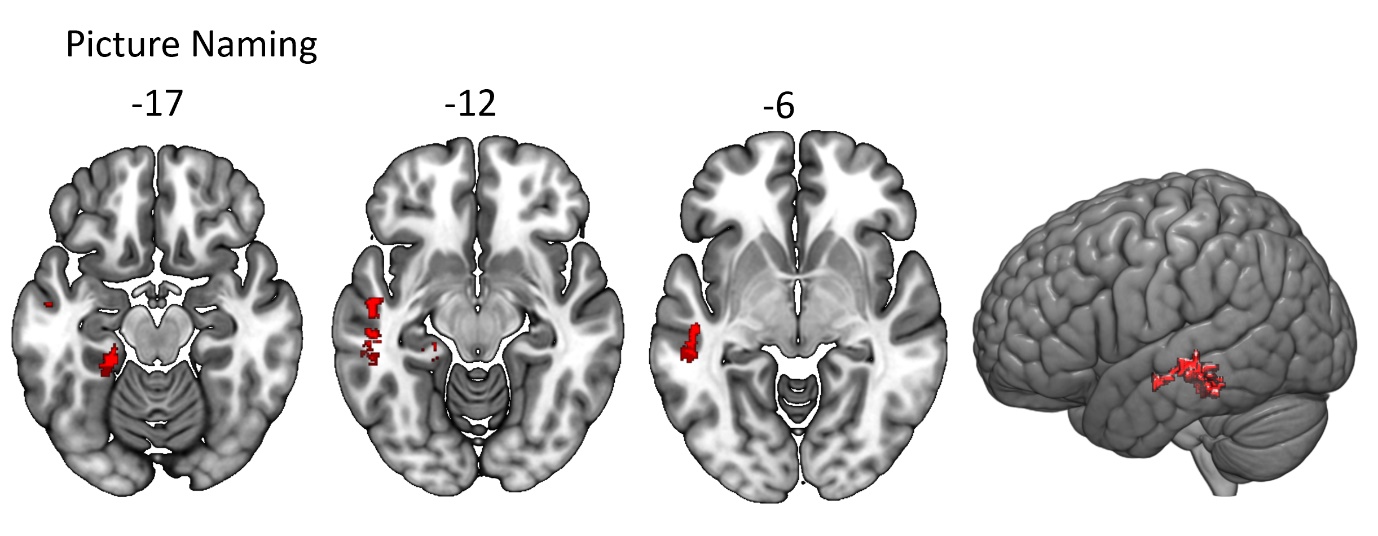


VLSM map of lesions associated with language deficits in the Picture Naming task, FDR corrected at p<0.05, displayed on a T1 anatomical template in MNI space.

SUPPLEMENTARY TABLE

|  |  |  |  | | **MNI Coordinates** | | | | | |  |
| --- | --- | --- | --- | --- | --- | --- | --- | --- | --- | --- | --- |
| **Task** | **Areas** | **Volume (mm^3^)** | | ***t* value** | | **X** | | **Y** | | **Z** | |
| Picture naming | ParaHippocampal Area 1 | 464 | 5.70 | | -22 | | -33 | | -17 | |  |
|  | Area STSd anterior | 99 | 5.75 | | -52 | | -25 | | -8 | |  |
|  | Area STSv posterior | 47 | 4.72 | | -50 | | -37 | | -12 | |  |

1. *VLSM lesions from Gesture Production identified in White Matter atlas*


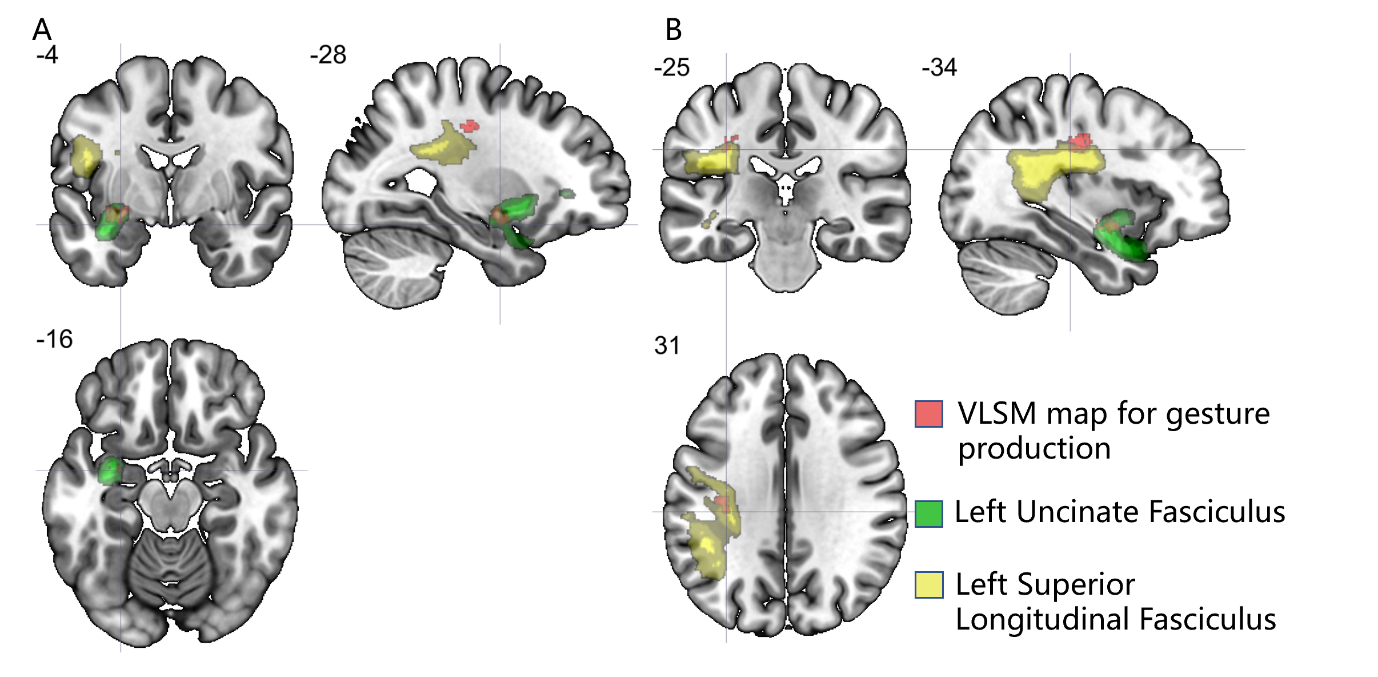


Figure 3. Anatomical locations of white matter lesions based on the Catani atlas of human brain connections. The significant regions for gesture production were enclosed in the Uncinate Fasciculus (A) and partly overlapped with the Superior Longitudinal Fasciculus (B). FDR corrected at p<0.05, displayed on a T1 anatomical template in MNI space.

1. *Settings used for our Voxel based lesion symptom mapping*

In the sections below, we outline methodological factors influencing our VLSM results.

1) Lesion delineation was automated, as opposed to manual: our choice of this method was to ensure our results were less prone to operator biases (Ashton et al., 2003; Gillebert et al., 2014; Wilke et al., 2011), whilst allowing the greater inclusion of patients due to time saved in delineating each patient’s lesion.

2) Patients were tested at early stages after stroke, to prevent the inclusion of atrophy in our lesion delineation, caused by post-stroke degeneration, known to occur at chronic stages (Lindberg et al., 2007; Gillebert et al., 2014; Gajardo-Vidal et al., 2018). However, it is noteworthy that previous lesion symptom mapping techniques investigating apraxia at early stages after stroke and using MRI have identified very similar results to ones reported at chronic stages (Manuel et al. 2013, Hoeren et al., 2014).

3) The use of covariates of no interest in this study (see methods), which included lesion size, and orientation in time and place, ensured, respectively, that i) our results were not caused by large lesions in a small number of patients (Lorca-Puls et al., 2018; Sperber and Karnath, 2018), and that ii) the behaviour of interest (i.e. Praxis) was not confounded by severe cognitive deficits caused by other clinical conditions, such as delirium.

4) The inclusion of an unselected, unbiased patient group, with either left or right hemisphere lesions, unlike previous studies in which only patients with left hemisphere lesions were selected (Buxbaum et al., 2014; Hoeren et al., 2014). This was based on work reporting bilateral representations of praxis functions, particularly relating to meaningless gesture imitation (Iacoboni et al. 2001, Buxbaum et al. 2007).

5) There are conflicting reports on the time course of recovery from apraxic deficits, which is the reason this was not controlled for in this study. Some studies report significant changes in praxis deficits in the first 8 months after stroke (Basso et al., 1987). Donkervoort et al. (2006) demonstrated little recovery after 2 months post-stroke. Bickerton et al. (2012) reported that approximately 50% of our cohort of patients tested in the Birmingham Cognitive Screening within the first three months after stroke, had recovered their praxis deficits when they were tested again at nine months. Stamenova et al. (2011) have suggested that the rates of recovery could differ between tasks.

6) Together with the use of a stringent statistical thresholding (FDR correction at p<0.05), the statistical maps we obtained appeared smaller and more distributed, but nevertheless still involved the left hemisphere, supporting the idea that this disorder affects a network of inter-connected regions, involved in praxis (Geschwind and Damasio, 1985).

*4. Subgroup VLSM Analyses:*

In the sections below, we provide sub-group analyses for each of the patient categories. Our behavioural data revealed comparable praxis scores in Right-, compared to Left-handed patients. In particular, the average score for: 1) Gesture Production in Right-handed patients was 10.01 (SD=2.2), and for Left-handed patients was 10.24 (SD 2.7); for Gesture Recognition in Right-handed patients was 4.91 (SD=1.1), and for Left-handed patients was 4.72 (SD=1.4); and for Meaningless Gesture Imitation in Right-handers was 9.01 (SD=2.4) and for Left-handed patients was 9.04 (SD=2.4). There were no significant differences between groups in each of the sub-scores (p>0.1).

The imaging data was re-analysed for these subgroups of patients. Due to the low number of patients who were Left-handed (N=34), and who had bilateral (N=9) strokes, there were no significant (FDR corrected at p<0.05), nor suprathreshold (uncorrected at p<0.005) lesioned voxels for those subgroups.

In the section below, we report the data for Right-handed patients, both lesioned hemisphere FDR- corrected (in subsection a.), and uncorrected (subsections b. and c.) at p<0.005 for left-, and right- hemisphere lesions, respectively.

a. Right Handed patients alone (both left, right and bilateral) hemispheres, FDR corrected at P<0.05

|  |  |  |  | | **MNI Coordinates** | | | | | |  |
| --- | --- | --- | --- | --- | --- | --- | --- | --- | --- | --- | --- |
| **Praxis Tasks** | **Areas** | **Volume (mm^3^)** | | ***t* value** | | **X** | | **Y** | | **Z** | |
| Gesture Production | Left Area STSd posterior | 43 | 5.36 | | -51 | | -34 | | -5 | |  |
|  |  |  |  | |  | |  | |  | |  |
|  | Left Caudate | 21 | 4.1 | | -10 | | 18 | | 6 | |  |
|  |  |  |  | |  | |  | |  | |  |
| Gesture Recognition | Left Area STSv posterior  Left Temporo-Parieto-Occipital Junction 1  Left Area dorsal 23 a/b (Posterior Cingulate)  Left Superior Longitudinal Fasciculus  Left Inferior Cerebellum  Left Inferior Occipito-Frontal Fasciculus (underlying V1)  Left Visual Area (V2)  Right Area 6 Anterior (Right dorsal premotor area PMd) | 2676  638  100  528  90  88  218  97 | 5.85  4.5  3.6  4.59  4.19  3.8  4.34  4.26  4.46  4.42 | | -55  -55  -53  -53  -1  -32  -37  -21  -14  25  21 | | -45  -41  -45  -46  -30  -33  -55  -82  -76  -3  10 | | -5  0  0  6  37  32  -47  0  -7  51  48 | |  |
| Meaningless Gesture Imitation | Left Inferior Occipito-Frontal Fasciculus (underlying extrastriate visual Cortex)  Left Inferior Longitudinal Fasciculus, adjacent and underlying PGi  Left paraHippocampal Area 2 | 130  76  45  42  23 | 5.6  4.11  5.13  5.14  4.64  4.81 | | -27  -30  -24  -34  -41  -31 | | -88  -78  -80  -69  -55  -39 | | -2  1  2  11  10  -14 | |  |


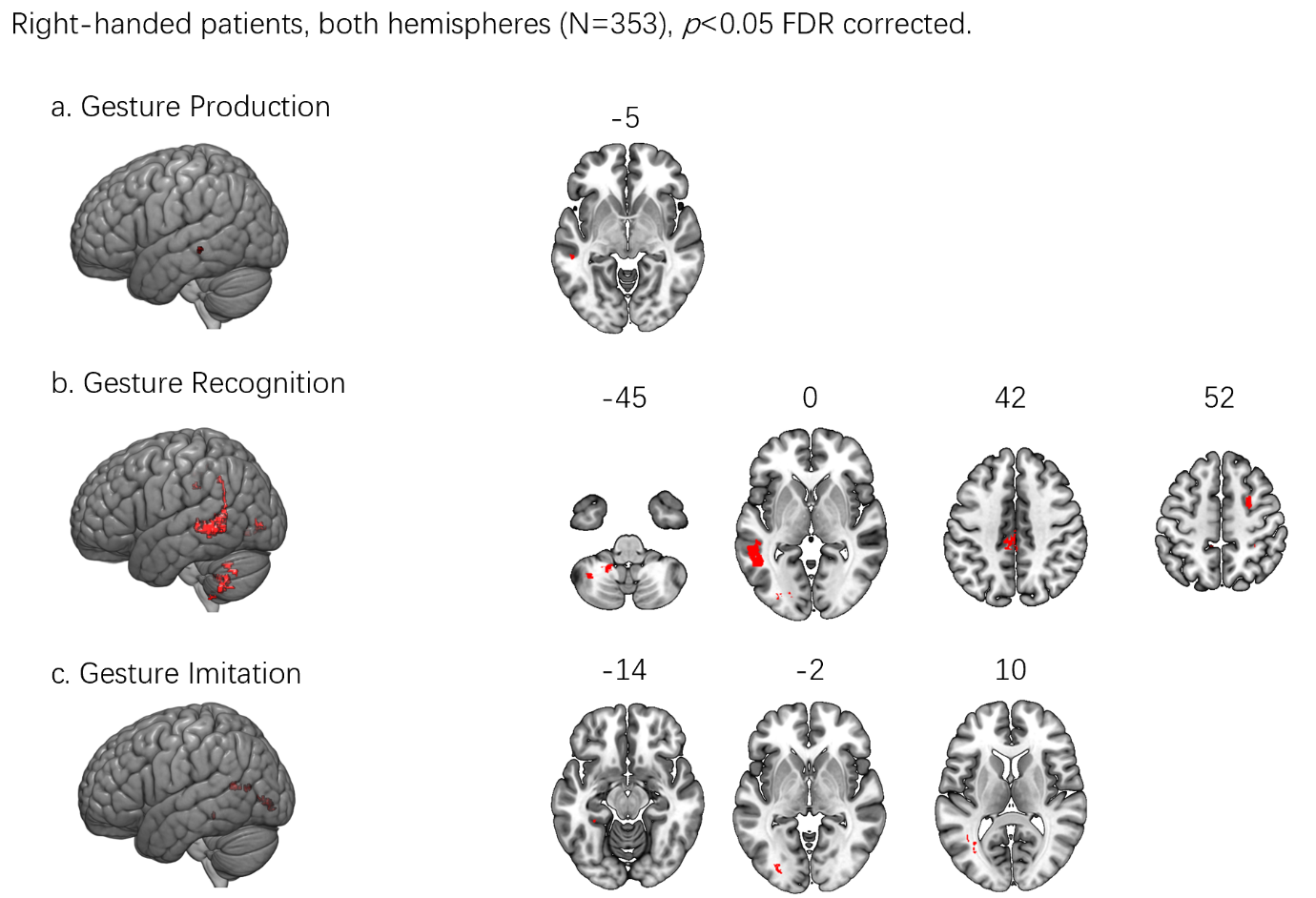


b. Left Hemisphere, right-handed, stroke patients (N=174), uncorrected at *P<0.005*

*No significant lesions identified with p<0.05 FDR correction. The data below are uncorrected at p<0.005.*

|  |  | |  |  | **MNI Coordinates** | | |
| --- | --- | --- | --- | --- | --- | --- | --- |
| **Praxis Tasks** | **Areas** | **Volume (mm^3^)** | | ***t* value** | **X** | **Y** | **Z** |
| Gesture Production | Left Superior Longitudinal Fasciculus  Left Inferior Occipito-Frontal Fasciculus (adjacent to Anterior Ventral Insula)  Left Caudate  Left Frontal Opercular Area 1 | | 1411  1462  242  129  31 | 3.91  4.56  4.21  3.32 | -30  -22  -17  -11  -46 | -18  24  23  21  -5 | 36  -7  -2  -1  7 |
|  |  | |  |  |  |  |  |
| Gesture Recognition | Left Frontal Opercular Area 1  Left Frontal Opercular Area 2  Left Frontal Opercular Area 4  Left Superior Longitudinal Fasciculus  Left Caudate  Left V1  Left V2 | | 2723  53  34  1593  44  95  466  54 | 4.04  2.96  3.37  3.86  3.04  2.74  2.95  2.74 | -45  -33  -35  -36  -18  -8  -18  -19 | -2  -6  10  -25  22  -97  -85  -99 | 8  16  10  36  8  -10  -14  4 |
| Gesture Imitation | Left Ventral Visual Complex  Left V4  Left V1  Left V2  Left Hippocampus/adjacent to left inferior occipito-frontal fasciculus | | 1271  528  42  963  47  358 | 2.74  4.3  2.69  3.4  2.74  4.17 | -34  -36  -8  -7  -10  -10  -32 | -65  -79  -97  -92  -78  -58  -41 | -17  -11  -10  1  -4  -5  -4 |
|  | Left Intraparietal Area 1 | | 275 | 3.51 | -28 | -56 | 34 |

*
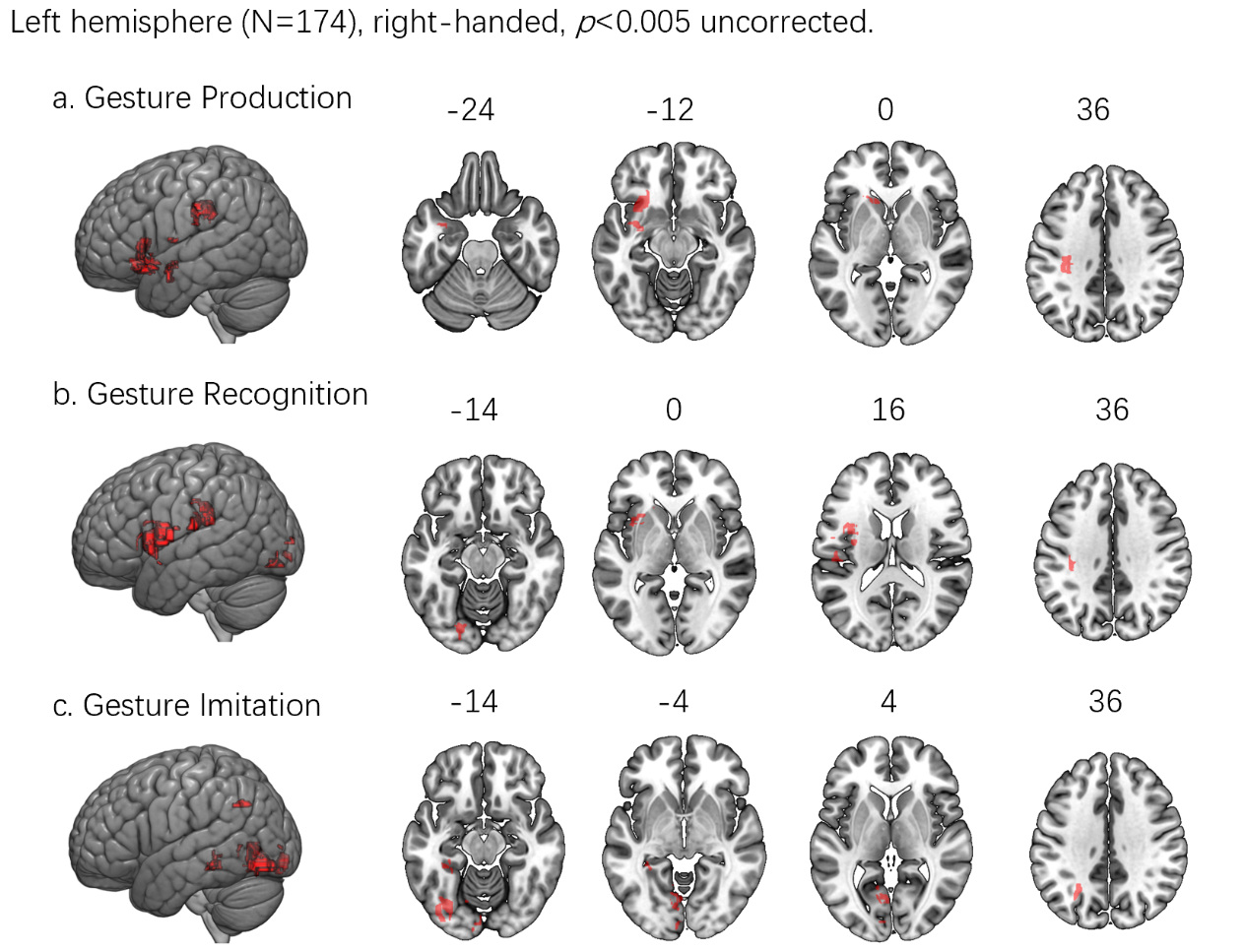
*

c. Right Hemisphere, right-handed, stroke patients (N=172), uncorrected at *P< 0.005*

*No significant lesions identified with p<0.05 FDR correction. The data below are uncorrected at p<0.005.*

|  |  | |  |  | **MNI Coordinates** | | |
| --- | --- | --- | --- | --- | --- | --- | --- |
| **Praxis Tasks** | **Areas** | **Volume (mm^3^)** | | ***t* value** | **X** | **Y** | **Z** |
| Gesture Production | No Suprathreshold voxels at P<0.005, uncorrected | |  |  |  |  |  |
|  |  | |  |  |  |  |  |
| Gesture Recognition | Right Area PFt  Right Opercular area PF  Right PFm Complex | | 1375  145  77 | 4.09  2.99  2.79 | 44  54  44 | -21  -15  -45 | 32  23  30 |
| Gesture Imitation | Right Lateral Belt Complex | | 267 | 4.44 | 43 | -28 | 11 |
|  |  | |  |  |  |  |  |
|  | Right Superior Longitudinal Fasciculus (underlying area AIP)  Right Area PGp | | 253  38 | 3.58  2.85 | 40  47 | -26  -73 | 28  18 |


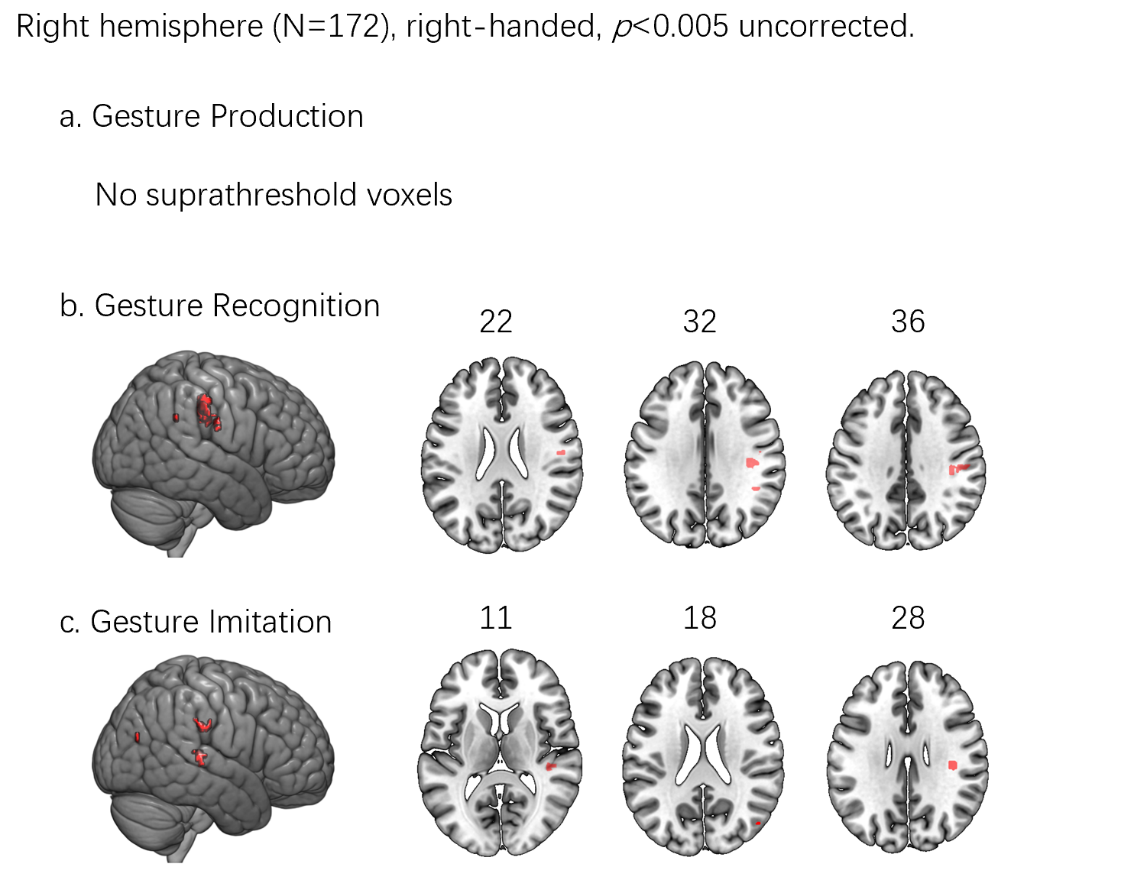


Additional References:

1. Basso A, Capitani E, Della Sala S, Laiacona M, Spinnler H. 1987. Recovery from ideomotor apraxia – a study on acute stroke patients. Brain. 110: 747-60.
2. Donkervoort M, Dekker J, Deelman B. 2006. The course of apraxia and ADL functioning in left hemisphere stroke patients treated in rehabilitation centres and nursing homes. Clinical Rehabilitation. 20(12): 1085-93.
3. Stamenova V, Black SE, Roy EA. 2011. A model-based approach to long-term recovery of limb apraxia after stroke. Journal of Clinical and Experimental Neuropsychology. 33(9): 954-71.
